# Effects of agricultural or gardening physical activity on cardiovascular disease and dementia-related markers via arterial stiffness, cognitive function, and cerebral white matter status: results from cross-sectional and interventional studies

**DOI:** 10.3389/fpubh.2025.1509528

**Published:** 2025-07-17

**Authors:** Masato Nishiwaki, Seiya Takada, Shotaro Otsuka, Hyuma Makizako, Harutoshi Sakakima, Naoto Shiomi, Satomi Ooba, Naoyuki Matsumoto, Kiyoshi Kikuchi

**Affiliations:** ^1^Faculty of Engineering, Osaka Institute of Technology, Osaka, Japan; ^2^Department of Orthopaedic Surgery, Graduate School of Medical and Dental Sciences, Kagoshima University, Kagoshima, Japan; ^3^RIKEN Center for Integrative Medical Sciences, Yokohama, Kanagawa, Japan; ^4^Faculty of Welfare and Health Science, Oita University, Dannoharu, Oita, Japan; ^5^Department of Physical Therapy, School of Health Sciences, Faculty of Medicine, Kagoshima University, Kagoshima, Japan; ^6^Department of Critical and Intensive Care Medicine, Shiga University of Medical Science, Otsu, Japan; ^7^Ooba Clinic for Neurosurgery and Headache, Oita, Japan; ^8^Faculty of Environmental Symbiotic Sciences, Prefectural University of Kumamoto, Kumamoto, Japan; ^9^Division of Brain Science, Department of Physiology, Kurume University School of Medicine, Kurume, Japan; ^10^Department of Neurosurgery, Kurume University School of Medicine, Kurume, Japan

**Keywords:** agriculture, cardiovascular diseases, cognition, vascular stiffness, white matter

## Abstract

**Background:**

Agricultural or gardening physical activity (AGPA) offers potential as a simple strategy to improve cardiovascular disease and dementia-related markers; however, the actual preventive effects remain unclear. Our objective was to investigate AGPA regarding related markers of cardiovascular disease and dementia using cross-sectional and interventional approaches.

**Methods and results:**

In Study 1, community-based older individuals were assessed, and 30 individuals who performed AGPA (AG group) and 30 1:1 age-, sex-, and objective activity-matched controls were cross-sectionally compared (mean age, 75 ± 6 y). Arterial stiffness (pulse wave velocity) was lower and hand-finger dexterity (pegboard test) was higher in the AG vs. control groups. The interventional results of Study 2 (secondary analysis of a randomized controlled trial) confirmed that consistent AGPA might regress white matter hyperintensities in older individuals.

**Conclusion:**

Our findings suggest that consistent AGPA *perse* may improve cardiovascular disease and dementia-related markers in older healthy individuals via arterial stiffness, cognitive function, and cerebral white matter status. This information could have major implications for integrated strategies for lifelong health.

**Secondary analysis of clinical trials (study 2):**

Study for Decreasing Depressive Symptoms and Increasing Memory Performance in the older adults (Trial registration number UMIN000018547, Date of registration 2015/08/07), URL: https://center6.umin.ac.jp/cgi-open-bin/ctr/ctr_view.cgi?recptno=R000021462.

## Introduction

Cardiovascular diseases (ischemic heart disease and stroke) are the world’s biggest killers (1). Deaths due to dementia have also increased dramatically, nearly four-fold since 2000. This became the fourth leading cause of death in high-income countries and is on track to overtake stroke as one of the top three (1). In fact, lifetime risks of stroke according to the Global Burden of Disease Study 2016 were higher in East Asia, including Japan (2). However, reduced mortality from cardiovascular diseases led to an increase in life expectancy in Japan over the past 30 years, and the increasing burdens of dementia and diabetes highlight areas needing focused attention and action (3). The general consensus is that multiple factors influence cardiovascular diseases and dementia but that modifiable risk factors, such as lifestyle, social networks, and physical activity (PA) appear particularly relevant and important factors amenable to intervention (4). Therefore, simple and effective PA strategies for preventing cardiovascular diseases and dementia are required.

Epidemiological studies have reported that engaging in agriculture or gardening is healthy behavior and is associated with low levels of cardiovascular disease, other chronic illnesses, and mortality rates (5–8). Consistent gardening may also exert preventive effects on brain aging, depression, and mood, and many of the world’s centenarians consistently engage in gardening as a hobby (6, 9, 10). Indeed, one study reported that daily gardening could reduce the incidence of dementia in future years (11). A scoping review evaluated the evidence supporting an impact of gardens and gardening on health (12). The authors reported multiple benefits involving social interaction, increased PA, and a reduction in depression and anxiety. However, observational findings, statistical reports, and subjective indices alone are insufficient for assessing the beneficial effects of agricultural or gardening PA (AGPA). Whether AGPA has a preventive impact on cardiovascular disease and dementia remains unclear, and further detailed study is needed.

The factors associated with AGPA that contribute to the prevention of cardiovascular disease and dementia remain controversial. In particular, PA is an integral part of AGPA, and PA is widely accepted as one of the most important factors in non-communicable disease prevention (13–16). However, distinguishing whether the preventive effects are actually related to AG-specific factors per se (e.g., soil, plant, or environmental factors, such as daylight and wind breezes) or to PA factors alone is difficult. These verifications can offer important new insights into the development of simple and effective strategies to prevent cardiovascular disease and dementia and also contribute to a better understanding of AGPA-induced physiological effects. However, to the best of our knowledge, no data are available regarding AGPA and the prevention of deleterious effects associated with aging and disease.

The primary aim of the present study was to investigate effects of AGPA on cardiovascular disease and dementia-related markers in older healthy individuals. In Study 1, we used cross-sectional data to examine the potential benefits of consistent AGPA on cardiovascular disease and dementia-related markers after considering measured PA levels. In Study 2, we used interventional data (secondary analysis of a randomized controlled trial), to examine whether consistent AGPA could restore cardiovascular disease and dementia-related markers. We hypothesized that (1) after considering measured PA levels, benefits of consistent AGPA are observed (Study 1) and that (2) consistent AGPA attenuates or at least partially restores cardiovascular disease and dementia-related parameters (Study 2).

## Methods

Study 1 was approved by the Human Ethics Committee at the Osaka Institute of Technology (206-7, 2018-1, and 2019-13) and Study 2 was approved by the Ethics Committee of the National Center for Geriatrics and Gerontology in Japan (#839). The procedures used in this study adhere to the tenets of the Declaration of Helsinki. Written informed consent was obtained from all participants before enrollment.

### Study 1: community-based pair-matched cross-sectional study

#### Participants

**Figure 1:**
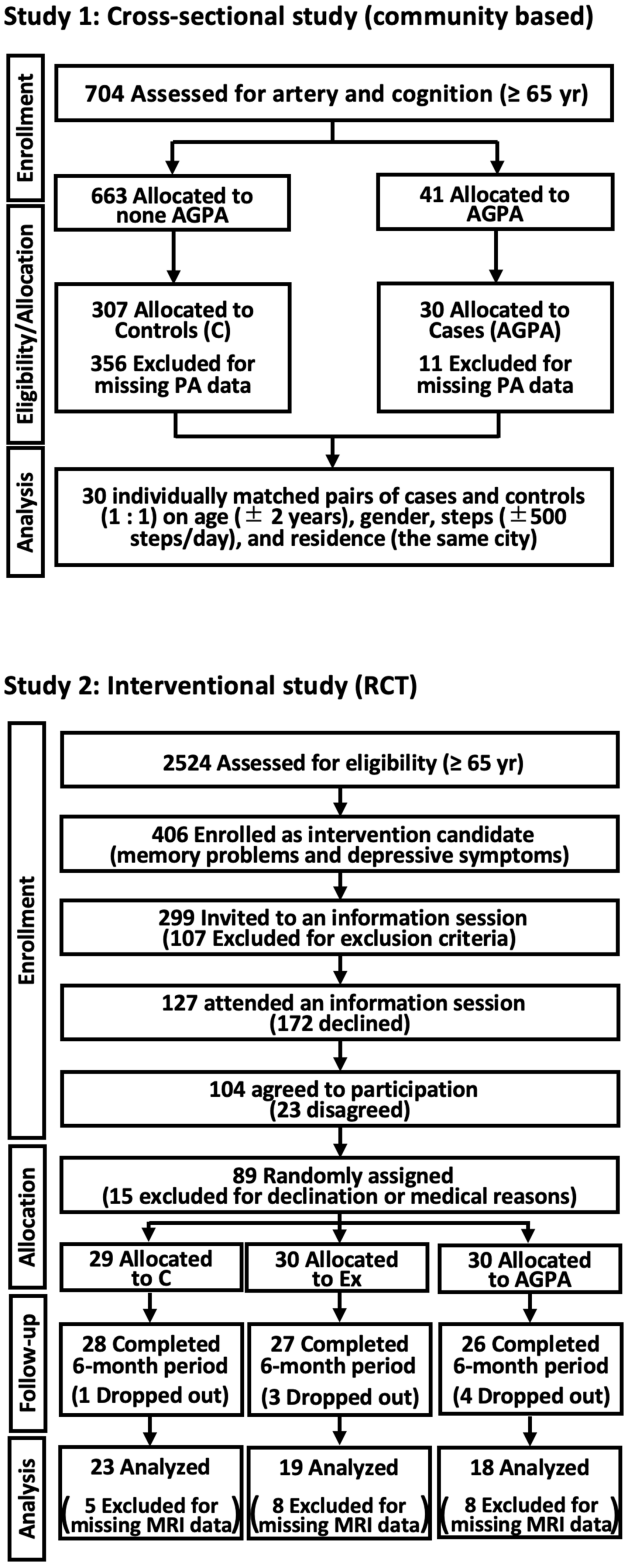
Flow diagram of study participation. C, controls; PA, physical activity; MRI, magnetic resonance imaging; RCT, randomized controlled trial; Ex, exercise group. To explore the preventive effects of agricultural or gardening physical activity (AGPA) on surrogate markers of cardiovascular disease and dementia, this study was comprehensively performed using two approaches (cross-sectional and interventional studies).

Potential participants were identified from data for 704 Japanese community dwellers ≥ 65 years old in the Osaka metropolitan area (male: *n* = 247; female: *n* = 457; age: 65–96 years; race: Asian) who were registered in health surveys by the Osaka Institute of Technology from September 2016 to January 2020 (Figure 1, Study 1) (17, 18). We extracted data for 41 participants who answered “yes” to a simple yes/no questionnaire confirming they were consistently engaged in occupational or hobby AGPA over 1 year. Of these 41 potential participants, AGPA data were available for 30 (11 had missing PA data). These 30 participants comprised the agriculture and gardening group (AG). A control group comprised 30 individuals who were 1:1 pair-matched with the AG group on the basis of gender (full match), age (± 2 years), and PA (± 500 steps/day) from among 307 registered participants in the health surveys with PA data in the same residential area. The data-matched pairs were then compared.

#### Main outcome measurements

The first main outcome was the assessment of arterial stiffness. We measured brachial-ankle pulse wave velocity (baPWV), heart-ankle pulse wave velocity (haPWV), and cardio-ankle vascular index (CAVI) using a semi-automated device (VS-1500AE/AN; Fukuda Denshi, Tokyo, Japan) (19, 20). Blood pressure and heart rate were assessed at the same time.

The second main outcome was the assessment of cognitive function. Hand-finger dexterity in both hands was assessed in triplicate for 15 s (each trial) using the pegboard test (T. K. K. 1,302; Takei Scientific Instruments, Tokyo, Japan), and the total number of displaced pegs was counted (17). Cognitive function was assessed using scores and response times from the Cognitive Assessment for Dementia, iPad version 2 (CADi2; Techno Project Japan, Shimane, Japan) (21). Scores range from 0 (low) to 10 (high), and scores ≤ 5 indicate suspected dementia (22).

#### Covariates

The height, weight, and body mass index (BMI) of the participants were assessed and measured by the researchers (TBF-410; Tanita, Tokyo, Japan). Age, gender, chronic diseases, smoking status, alcohol consumption, marital status, academic background, sports activities, and agriculture or gardening status were assessed from questionnaires and in-person interviews. Additionally, the Geriatric Depression Scale 15 (GDS-15) comprising 15 questions describing depressive symptoms (scores range from 0 to 15), with higher scores indicating worse depression (23), sit-and-reach test (T-283 device; Toei Light, Tokyo, Japan), handgrip strength (T. K. K. 5,001 Grip-A; Takei Scientific Instruments), and 6-meter walk test were assessed (17–19). Total PA and steps for 1 week were detected by an activity monitor (Lifecorder PLUS; Suzuken, Aichi, Japan) under sealed conditions (uninformed measured values for the participants). General criteria of wearing the monitor for > 10 h, no non-wear time ≥ 60 consecutive minutes, and conversion to metabolic equivalents were applied in the data analyses (18, 24).

### Study 2: randomized controlled trial (RCT)

#### Participants and interventions

This study was a prespecified interventional study (secondary analysis) of a single-blind RCT performed in Japan between 2016 and 2017 for community-based older adults ≥ 65 years with depressive symptoms and memory difficulties, in the Aichi metropolitan area (UMIN000018547) (25). The details of the protocol, inclusion criteria, and main findings have been published (26). Briefly, the randomization procedure was performed by a researcher who was unaware of the aims of the study, and computer-generated random number allocation was used to divide participants into three groups (1:1:1), as follows: educational control group (control group); exercise intervention group; and AGPA intervention group (AGPA). Each group underwent a 20-week intervention, and data were compared before and 12 months after the interventions. Although available data for brain MRI were limited, data for 60 (controls, *n* = 23; exercise intervention, *n* = 19; AGPA, *n* = 18) older adults were analyzed (Figure 1, Study 2). Participants in the control group attended two 90-min education classes (i.e., traffic safety and disaster prevention) that experts considered less likely to influence the study outcomes during the intervention period. A multi-component exercise program was performed in the exercise intervention group, comprising weekly 90-min sessions of aerobic exercise, muscle strength training, postural balance retraining, and dual-task training (PA and cognitive tasks). The program for AGPA entailed weekly 60- to 90-min sessions comprising nature-based group activities. The program comprised crop-related activities, such as cultivating, growing, and harvesting. AGPA participants also engaged in gardening activities that included group planting (known as Yoseue-style bonsai), which involved a combination of different plant varieties or shapes and planting flowers in a public garden.

#### Outcomes measurements and data analysis

Whole-brain magnetic resonance imaging (MRI) was performed using a 3-T system (Tim Trio; Siemens Healthcare, Erlangen, Germany), and white matter hyperintensity (WMH) volumes in the corpus callosum, and periventricular and deep and subcortical regions were assessed using brain anatomical analysis using diffeomorphic deformation software version 4.3,^1^ as previously described (27).

#### Covariates

As previously described (25, 26), baseline participant characteristic data were aggregated. To evaluate the effect of the interventions, the results of the GDS-15 and Mini-Mental State Examination; tablet versions of the trail-making test; concentrations of cortisol, insulin-like growth factor-1 (IGF-1), and brain-derived neurotrophic factor (BDNF); handgrip strength; walking speed; and the walking test were analyzed, as previously reported in detail (25, 26).

### Statistical analysis

The results are presented as mean ± standard deviation. In Study 1, continuous data were analyzed using the independent t-test and analysis of covariance (ANCOVA), which included the covariates. Differences in non-parametric variables were analyzed by the Mann–Whitney U test. One-way ANOVA for baseline characteristics, two-way repeated-measures ANOVA followed by the Bonferroni method for changes in all measured parameters, and the Kruskal–Wallis test were used for the analyses in Study 2. In all statistical analyses, pairwise deletion methods (available-case analysis) were used if missing data were included in the main outcomes or covariates. Data were statistically analyzed using SPSS version 25.0 J (IBM SPSS Japan, Tokyo, Japan), Excel Statistics 2015 (Social Survey Research Information, Tokyo, Japan), and Prism version 9.2.0 (GraphPad Software, San Diego, CA, United States). As previously described (17–19, 24), coefficients of variation as a measure of reproducibility for our all-measurement parameters on two separate days were < 10%. Effect size and statistical power (1 − *β*) were calculated using G*Power 3. *p* < 0.05 was considered statistically significant.

## Results

### Study 1: community-based pair-matched cross-sectional study

**Figure 2:**
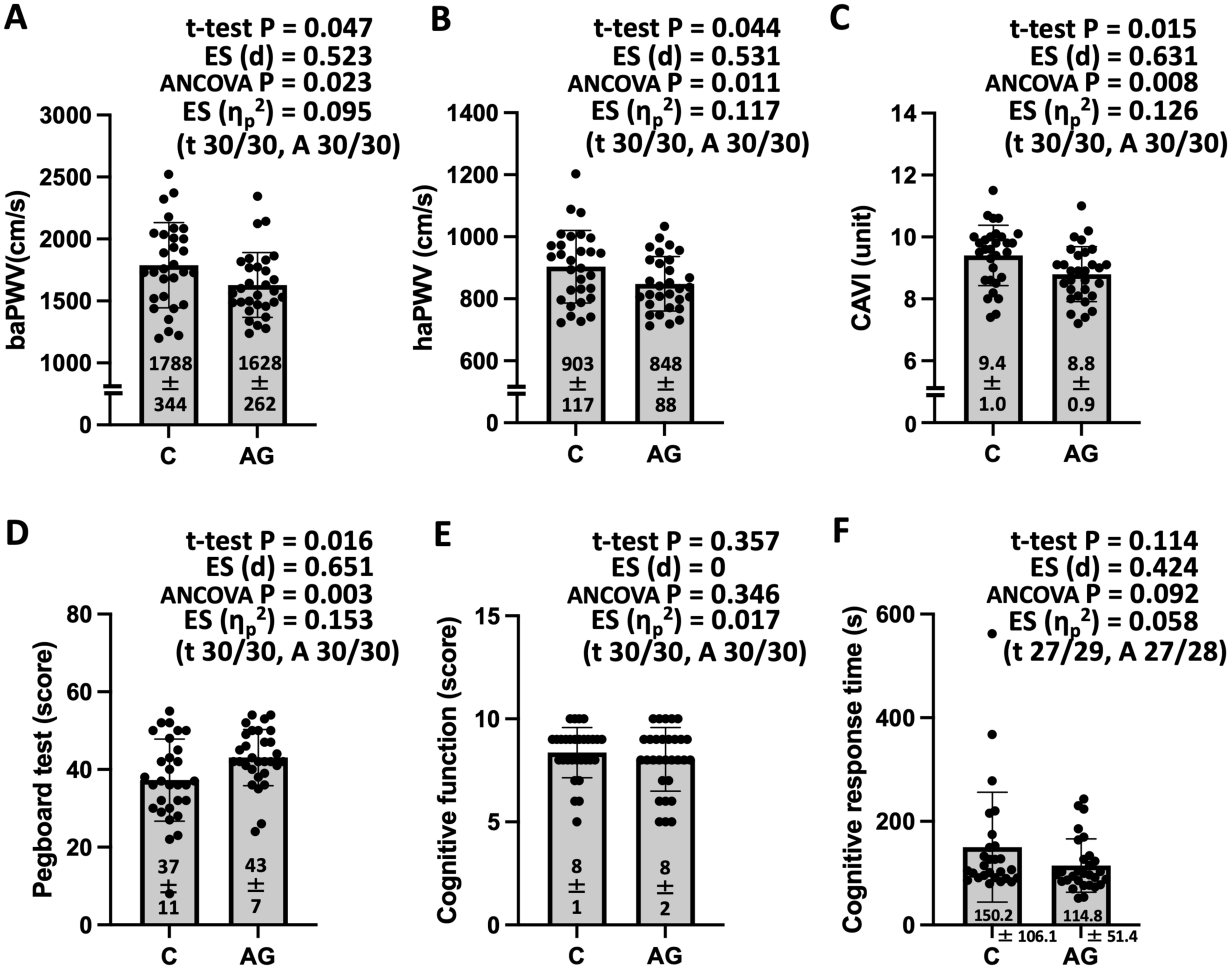
Arterial stiffness **(A–C)**, dexterity **(D)**, and cognitive function **(E,F)** for participants in the community-based pair-matched cross-sectional study (Study 1). baPWV, brachial-ankle pulse wave velocity; haPWV, heart-ankle pulse wave velocity; CAVI, cardio-ankle vascular index; ES, effect size; η_p_^2^, ANCOVA, analysis of covariance (with age, sex, body mass index, mean blood pressure, sports activities, and current smoker status as covariates). The values shown in parentheses are the numbers of analyzed participants in the control (C) and agriculture and gardening physical activity (AGPA) groups for the t-test (t) and ANCOVA (A). Data are expressed as mean ± standard deviation. Study 1 showed significantly lower arterial stiffness and higher dexterity in the agricultural or gardening physical activity (AG) group compared with the control (C) group.

In addition to pair-matched age, gender, and objective PA data, no significant differences were observed in almost all participant characteristics between the two groups (Table 1). Nevertheless, with or without adjusting the outcomes for the covariates, baPWV, haPWV, and CAVI, as indices of arterial stiffness, were significantly lower in the AG than in the control groups (Figures 2A–C). A significantly higher pegboard score and a tendency toward shorter response time during cognitive testing were also found in the AG group compared with the control group (Figures 2D–F).

**Table 1:** Characteristics of community-based pair-matched cross-sectional study participants (Study 1).

Parameters	C group			AG group			*p*-value
Participants, n (men/women)	*n* = 30 (11/19)			*n* = 30 (11/19)			–
Age, year	75	±	6	75	±	6	1.000
Height, cm	152.3	±	8.5	153.1	±	8.0	0.695
Weight, kg	54.6	±	9.5	53.7	±	9.2	0.718
BMI, kg/m^2^	23.4	±	2.8	22.9	±	3.3	0.499
Body fat, %	23.5	±	7.2	24.0	±	11.3	0.849
Waist circumference, cm	83.9	±	8.5	82.4	±	18.0	0.676
Sit-and-reach	29.0	±	9.8	33.8	±	10.7	0.079
Handgrip strength, kg	24.8	±	7.6	24.9	±	8.3	0.933
Walking speed, m/s	1.55	±	0.32	1.75	±	0.44	0.049
Heart rate, beats/min	71	±	10	70	±	10	0.775
Systolic BP, mmHg	142	±	17	142	±	19	0.994
Diastolic BP, mmHg	82	±	9	83	±	9	0.695
Steps, steps/day	5,373	±	3,071	5,366	±	3,050	0.993
Time spent in light activity, min/day	43.0	±	18.8	42.7	±	20.2	0.966
Time spent in moderate, min/day	10.6	±	13.0	12.8	±	13.0	0.526
Time spent in Vigorous, min/day	0.49	±	0.63	0.79	±	1.22	0.227
Sports habits, n (%)	21 (70.0)			19 (63.3)			0.590
Geriatric depression scale 15, scores	3	±	2	3	±	3	0.936
Anti-hypertensive medication, n (%)	16 (53.3)			14 (46.7)			0.986
Anti-hyperlipidemic medication, n (%)	6 (20.0)			9 (30.0)			0.680
Current smokers, n (%)	3 (10.0)			2 (6.7)			0.643
Alcohol consumption	*n* = 29			*n* = 30			0.276
Non-drinker, n (%)	15 (51.7)			21 (70.0)			–
Non-daily drinker, n (%)	10 (34.5)			4 (13.3)			–
Daily drinker, n (%)	4 (13.8)			5 (16.7)			–
Marital status	*n* = 27			*n* = 29			0.642
Married, n (%)	21 (77.8)			24 (82.8)			–
Loss, n (%)	6 (22.2)			5 (17.2)			–
Educational attainment	*n* = 28			*n* = 28			0.583
< 9 years, n (%)	8 (28.6)			11 (39.3)			–
10–12 years, n (%)	12 (42.9)			9 (32.1)			–
≥ 13 years, n (%)	8 (28.6)			8 (28.6)			–

BMI, body mass index; BP, blood pressure; C group, paired matched control group; AG group, agriculture and gardening group; Data are expressed as means ± SD. *p* values are expressed as the results of Independent *t*-tests or Mann–Whitney U tests.

### Study 2: RCT involving older adults

No significant differences in baseline characteristics were observed between the groups (Table 2). After the intervention, GDS-15 scores and cortisol concentrations decreased significantly in all groups. Although no significant interactions in the two-way ANOVA were found, walking speed [effect size (dz) = 0.85] and 2-min walking distance [effect size (dz) = 0.58] improved significantly only in the exercise intervention group. The IGF-1 concentration [effect size (dz) = 0.44] increased significantly only in the AGPA group (Table 3).

**Table 2:** Baseline physiological characteristics of RCT (Study 2).

Parameters	C group			Ex group			AGPA group			*p* value
Participants, n (men/women)	*n* = 23 (12/11)			*n* = 19 (8/11)			*n* = 18 (13/5)			–
Age, year	72	±	5	73	±	6	74	±	6	0.397
Height, cm	158.9	±	11.2	157.1	±	9.0	160.6	±	7.6	0.552
Weight, kg	55.9	±	11.0	55.2	±	10.2	61.6	±	8.5	0.113
BMI, kg/m^2^	22.2	±	3.2	22.3	±	3.7	24.0	±	4.1	0.244
Systolic BP, mmHg	136	±	22	141	±	25	133	±	25	0.636
Diastolic BP, mmHg	77	±	13	80	±	12	77	±	12	0.692
Medical history										
Hypertension, n (%)	*n* = 12 (52.2)			*n* = 7 (36.8)			*n* = 7 (38.9)			0.554
Diabetes mellitus, n (%)	*n* = 5 (21.7)			*n* = 4 (21.1)			*n* = 5 (27.8)			0.648
Steps, steps/day	5,547	±	2,135	5,787	±	2,576	5,874	±	3,927	0.932
Education, year	12	±	2	12	±	2	12	±	3	0.881

C, educational control group; Ex, exercise intervention group; AGPA, agricultural or gardening physical activity (AGPA) intervention group; BMI, body mass index; BP, blood pressure. Data are expressed as means ± SD. P values are expressed as the results of one-way ANOVA.

**Table 3:** Physiological and psychological parameters before and after the intervention period.

Parameters	C group						Ex group						AGPA group						Int. P
	Pre			Post			Pre			Post			Pre			Post			
GDS-15, Score	6.4	±	2.4	4.7	±	3.1*	6.7	±	2.2	4.8	±	2.6*	6.6	±	1.7	4.4	±	3.4*	0.910
MSSE, Score	28.3	±	1.7	28.1	±	1.7	28.4	±	1.6	28.2	±	2.3	27.1	±	3.0	26.8	±	3.1	0.958
TMT-A, s	19.2	±	2.7	20.7	±	7.8	19.4	±	5.5	19.9	±	5.7	23.3	±	7.3	24.1	±	9.2	0.853
TMT-B, s	35.6	±	13.0	36.5	±	15.5	29.9	±	9.1	34.5	±	14.9	54.1	±	26.1	57.8	±	67.0	0.926
Cortisol, μg/dL	10.1	±	5.4	7.4	±	3.2*	10.2	±	5.5	7.1	±	3.0*	10.9	±	4.2	7.2	±	3.4*	0.846
IGF-1, ng/mL	101.9	±	25.1	102.4	±	23.1	94.1	±	24.5	93.2	±	28.5	94.2	±	31.2	104.1	±	31.1*	0.200
BDNF, ng/dL	19.2	±	7.5	16.4	±	11.2	18.9	±	8.0	18.5	±	10.6	16.0	±	7.8	16.8	±	10.2	0.661
Strength, kg	29.8	±	10.0	28.8	±	10.7	25.9	±	7.0	25.9	±	8.5	29.8	±	7.7	26.8	±	6.2*	0.106
Walking speed, m/s	1.32	±	0.21	1.34	±	0.23	1.30	±	0.17	1.38	±	0.16*	1.23	±	0.17	1.23	±	0.19	0.216
Two-min walk., m	155.5	±	20.2	157.0	±	22.6	149.1	±	23.9	158.4	±	24.5*	140.0	±	29.9	144.2	±	29.3	0.365

C, educational control group; Ex, exercise intervention group; AGPA group, agricultural or gardening physical activity intervention group; Int. P, interaction *p* values; Pre, before intervention; Post, after intervention; GDS-15, geriatric depression scale-15; MMSE, mini-mental state examination; TMT, trail-making test; IGF-1, insulin-like growth factor-1; BDNF, serum brain-derived neurotrophic factor; Strength, handgrip strength, Two-min walk., two-min walking test. *p < 0.05 vs. each Pre (simple main effect). Data are expressed as means ± SD.

A higher participant rate of reduced corpus callosum WMH volume was observed in both the AGPA [38.9%, 7/18; effect size (r) = −0.24] and exercise intervention groups [52.6%, 10/19; effect size (r) = −0.37] compared with the control group (17.4%, 4/23; *p* = 0.056). Moreover, the participant rate of low PVH volume was slightly higher in the AGPA group [22.2%, 4/18; effect size (r) = −0.01] and notably higher in the exercise intervention group [36.8%, 7/19; effect size (r) = −0.16] compared with the control group (17.4%, 4/23; *p* = 0.485). The participant rate of reduced DSWMH volume was similar in all groups [controls: 21.7%, 5/23; exercise intervention group: 21.1%, 4/19, effect size (r) = 0.01; AGPA: 22.2%, 4/18, effect size (r) = −0.01; *p* = 0.996].

## Discussion

The primary purpose of the study was to investigate effects of AGPA on cardiovascular disease and dementia-related markers via arterial stiffness, cognitive function, and cerebral white matter status using cross-sectional and interventional approaches. First, in Study 1, lower arterial stiffness, higher dexterity, and a tendency toward higher cognitive function were found in the AGPA group compared with the control group after considering daily PA levels via pair-matched comparison. Moreover, Study 2 confirmed that consistent AGPA might lead to regression of WMH volume, similar to other forms of exercise. To the best of our knowledge, our results suggest that consistent AGPA perse may improve cardiovascular disease and dementia-related markers in older healthy individuals.

Observational data show that people engaged in agriculture or gardening are healthier and live longer than those who are not engaged in these activities (5–8); however, PA might be the sole contributor to this status. Nevertheless, the results of Study 1 indicated that lower arterial stiffness, higher dexterity, and a tendency toward higher cognitive function were found in the AGPA group compared with the control group, using an objective PA pair-matched method. Similarly, Kingsley et al. reported that participants who engaged in ≥ 150 min/week of vigorous gardening had lower cardiometabolic risk scores, waist circumference, diastolic blood pressure, and triglyceride concentrations compared with those who did not engage in vigorous gardening (28). Increased arterial stiffness is widely accepted as an independent risk factor for future cardiovascular disease (29) and indicates a decline in the buffering capacity of relatively large arteries to pulsatile systolic pressure and conversion of pulsatile cardiac ejection into continuous blood flow through the capillary beds (30). Hand-finger dexterity test scores are strongly related to cognitive function (31). The CADi2 cognitive test is a useful tool for assessing dementia in Japanese populations, and the scores correlate well with those of the Mini-Mental State Examination (22). Therefore, our findings indicate that consistent AGPA can prevent cardiovascular disease and dementia-related markers after considering PA levels.

It is unclear that the physiological mechanisms of measured parameter differences with AGPA. However, several possibilities are considered. First, as AGPA-specific factors, hand and finger movements are used in activities such as planting seedlings or flowers, and the associated fine-movement tasks may increase dexterity and cognitive function. A previous study indicated that dual-task exercise training increased cognitive function more than did exercise alone (32). AGPA involves dual-task exercise, which is not present with cycling exercise. As a result, AGPA was associated with a higher score of cognitive function. Furthermore, improved mental health might result from soil, plant, and environmental factors (daylight or breezes) encountered during AGPA (6). Indeed, previous studies have reported that exposure to green space is associated with mental health, self-reported health, and well-being (33, 34).

Second, favorable results can result from the specific mode and type of AGPA. AGPA is a systemic activity that requires both upper and lower limbs. Higher muscle participation increases blood flow, especially in the upper limbs, and NOx concentrations and hyperfibrinolysis in the endothelium may increase more readily with AGPA. Indeed, a cohort study of 80,306 British adults revealed a significant reduction in cardiovascular disease mortality with swimming, racquet sports, and aerobics; however, no significant associations were found for cycling, running, or football (35). These findings suggest that exercise using both the upper and lower limbs is a major factor in preventing cardiovascular disease mortality. Another study demonstrated that interval exercise increased the internal carotid artery shear rate more than the equivalent work volume of continuous exercise, and the authors reported that interval exercise may be more effective and result in greater improvements in cardiovascular function. This relates to a decrease in the risk of cardiovascular diseases with AGPA compared with continuous exercise (36). AGPA is almost a non-continuous exercise, similar to various types of interval or circuit training, and this might contribute to the favorable effects of AGPA on cardiovascular status and cognitive function.

Finally, as general PA factors, aerobic exercise is well known to increase blood flow and shear stress, and repetitive changes in these parameters trigger vasodilator release and reductions in arterial stiffness (30, 37). Blood flow and shear stress also produce an increase in t-PA, but not in PAI-1, in endothelium (38). Change in the balance between tissue-type plasminogen activator and PAI-1 might thus increase plasmin and induce hyperfibrinolysis. Previous studies have indicated that such hyperfibrinolysis might induce activation of BDNF and, thereby, improve neurological symptoms (39). Accordingly, these combined effects of specific and general factors in AGPA might help prevent cardiovascular disease and dementia-related markers.

Although consistent PA improves arterial stiffness and cognitive function (30, 32), WMH often progresses over time and usually does not improve (40). However, recent studies have identified the possibility of WMH regression (41). The results of Study 2 indicated that consistent AGPA as well as cycling exercise may regress WMH, particularly in the corpus callosum. Interestingly, in contrast to the exercise intervention group, the AGPA group did not show significant improvement in the physical fitness parameters. These results may indicate that specific factors in AGPA as well as general PA factors have preventive effects on WMH status. The corpus callosum is the largest white matter tract in the brain and is considered important for communication and communication between the cerebral hemispheres (42). Therefore, regression of WMH in the corpus callosum would protect against dementia and age-related declines in cognitive and motor functions (42). Data from animal studies (Supplementary file; Supplementary Figure S1; Supplementary text) also support the idea that consistent preconditioning PA induces extensive neurovascular protective effects against ischemic and hemorrhagic stroke. Similarly, a human study reported that consistent PA may not only prevent stroke and dementia but also reduce disability after stroke (43), supporting our data. Therefore, consistent long-term AGPA could induce preventive effects in cardiovascular disease and dementia, as shown by our results in the cross-sectional and interventional studies.

The United Nations adopted 17 sustainable development goals (SDGs) (44, 45). Regular AGPA can certainly promote good health and well-being (Goal 3). Furthermore, the adoption of AGPA might contribute to food (Goal 2), clean water (Goal 6), sustainable city development (Goal 11), sustainable consumption and production patterns (Goal 12), and countermeasures against climate and environmental problems (Goals 13 and 15). Therefore, promotion of consistent AGPA, not merely PA, may be important, particularly in high-income countries, and offers an opportunity for individuals to feel closer to and think about health, food, climate, and environmental problems. Promotion of consistent AGPA thus contributes to reaching sustainable development goals.

This study has several important potential limitations. First, group allocation was performed by self-reported AGPA engagement. Selection bias might be present although the study used a data extract from relatively modest cohort sizes and several sensitivity analyses. The AGPA and control groups may also have intrinsic lifestyle differences beyond measured covariates. Although comparisons are conducted within the same cohort living in the same area, our cross-sectional results may be affected by unmeasured confounders, especially dietary habits. Additionally, there were missing data for some covariate parameters (i.e., marital status, alcohol use, and educational status), and listwise deletion analyses were thus used. Second, the data comprised both leisure and occupational AGPA, and the focus was Japanese older adults. This study evaluated the hypothesis that AGPA could induce physiological preventive effects in cardiovascular disease and dementia surrogate markers, and our findings are supported by international observational data (5, 7–9, 11). However, agricultural practices and regional environments may differ markedly in other countries compared with Japan. Recent epidemiological evidence also indicates that while higher leisure-time PA is associated with reduced cardiovascular events, higher occupational PA is associated with increased risks and that these are independent of each other (46). Whether status differences in AGPA would alter the favorable effects on cardiovascular disease and dementia remains unclear. Based on our results, further large-scale, long-term interventional studies are warranted to elucidate the multifaceted effects of AGPA in the prevention of cardiovascular disease and dementia.

## Conclusion

This study aimed to comprehensively investigate the effects of AGPA on cardiovascular disease and dementia-related markers using cross-sectional and interventional approaches. Our results indicate that (1) consistent AGPA can prevent cardiovascular disease and dementia-related markers after considering PA levels, and that (2) consistent long-term AGPA might induce preventive effects in cardiovascular disease and dementia. Our findings suggest that consistent AGPA *perse* may improve cardiovascular disease and dementia-related markers in older healthy individuals via arterial stiffness, cognitive function, and cerebral white matter status. This information could have major implications for integrated strategies for lifelong health as simple and effective methods.

## Data Availability

The raw data supporting the conclusions of this article will be made available by the authors, without undue reservation.

## Footnotes

^1^ https://www.shiga-med.ac.jp/hqbioph/iBaad/page0.html
