# Supplementary material for: Effects of agricultural or gardening physical activity on cardiovascular disease and dementia-related markers via arterial stiffness, cognitive function, and cerebral white matter status: results from cross-sectional and interventional studies
Source: Front Public Health. 2025 Jul 17;13:1509528. doi: 10.3389/fpubh.2025.1509528 (PMC12311633; doi:10.3389/fpubh.2025.1509528)
Supplement: Supplementary file 1 [file Presentation_1.pdf]

Supporting information file

This document contains figures and text for supplementary animal experiments:

Masato Nishiwaki[1*#], Seiya Takada[2,3], Shotaro Otsuka[4], Hyuma Makizako[5], Harutoshi Sakakima[5], Naoto Shiomi[6], Satomi Ooba[7], Naoyuki Matsumoto[8], and Kiyoshi Kikuchi[9, 10*#]

[1] Faculty of Engineering, Osaka Institute of Technology, Osaka, Japan

[2] Department of Orthopaedic Surgery, Graduate School of Medical and Dental Sciences, Kagoshima University, Kagoshima, Japan

[3] RIKEN Center for Integrative Medical Sciences, Yokohama, Kanagawa, Japan

[4] Faculty of Welfare and Health Science, Oita University, Dannoharu, Oita, Japan

[5] Department of Physical Therapy, School of Health Sciences, Faculty of Medicine, Kagoshima University, Kagoshima, Japan

[6] Department of Critical and Intensive Care Medicine, Shiga University of Medical Science, Otsu, Japan

[7] Ooba Clinic for Neurosurgery and Headache, Oita, Japan

[8] Faculty of Environmental Symbiotic Sciences, Prefectural University of Kumamoto, Kumamoto, Japan

[9] Division of Brain Science, Department of Physiology, Kurume University School of Medicine, Kurume, Japan

[10] Department of Neurosurgery, Kurume University School of Medicine, Kurume, Japan

[*] Corresponding author:
Kiyoshi Kikuchi, DDS, MD, PhD
Division of Brain Science, Department of Physiology, Kurume University School of Medicine, 67 Asahi-machi, Kurume, Fukuoka, 830-0011, Japan
Phone: (+81) 942-31-7542, Fax: (+81) 942-31-7695
Email: kikuchi_kiyoshi@kurume-u.ac.jp

# These authors contributed equally to this work.

Title: Preventive effects of regular preconditioning exercise on hemorrhagic and ischemic strokes in animals (supplementary animal experiments)

Contents:

Fig. S1 Time course of the animal experiments

Fig. S2 Preconditioning exercise and hemorrhagic stroke in mice

Fig. S3 Preconditioning exercise and ischemic stroke in rats

Text: Methods and results of the animal experiments

Fig. S1 Time course of the animal experiments

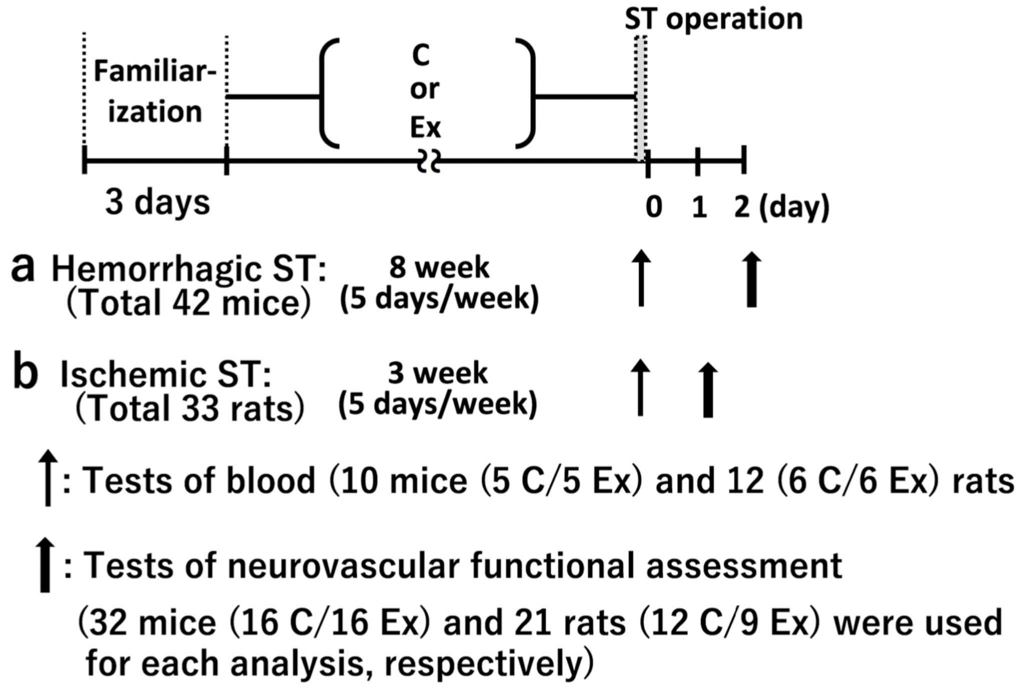

C, controls; Ex, exercise group; ST, stroke

Fig. S2 Preconditioning exercise and hemorrhagic stroke in mice

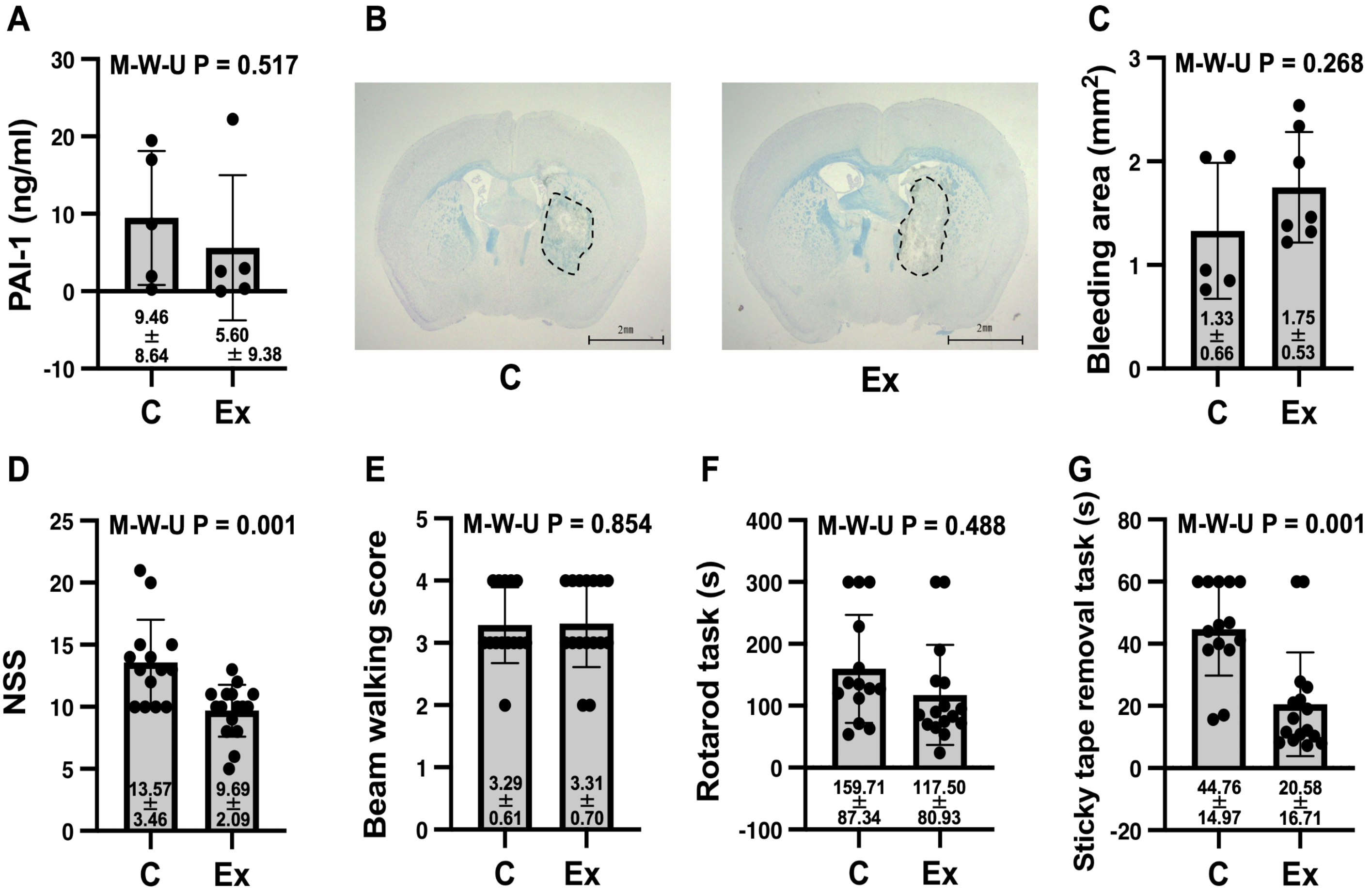

A) Concentrations of active plasminogen activator inhibitor-1 (PAI-1) tended to be lower in the exercise (Ex) group compared with the control (C) group. B) Representative Klüver–Barrera-stained cerebral sections from the C and Ex groups. C) Bleeding areas tended to be larger in the Ex group compared with the C group. D–G) NSS (D), beam walking score (E), rotarod task (F), and sticky tape-removal task (G). Data are expressed as mean ± standard deviation.

Fig. S2 shows that regular preconditioning exercise before hemorrhagic stroke significantly improved performance in neurological severity scores (NSS) and the sticky tape-removal task compared with the no-exercise control condition.

M-W-U, Mann-Whitney U test.

Fig. S3 Preconditioning exercise and ischemic stroke in rats

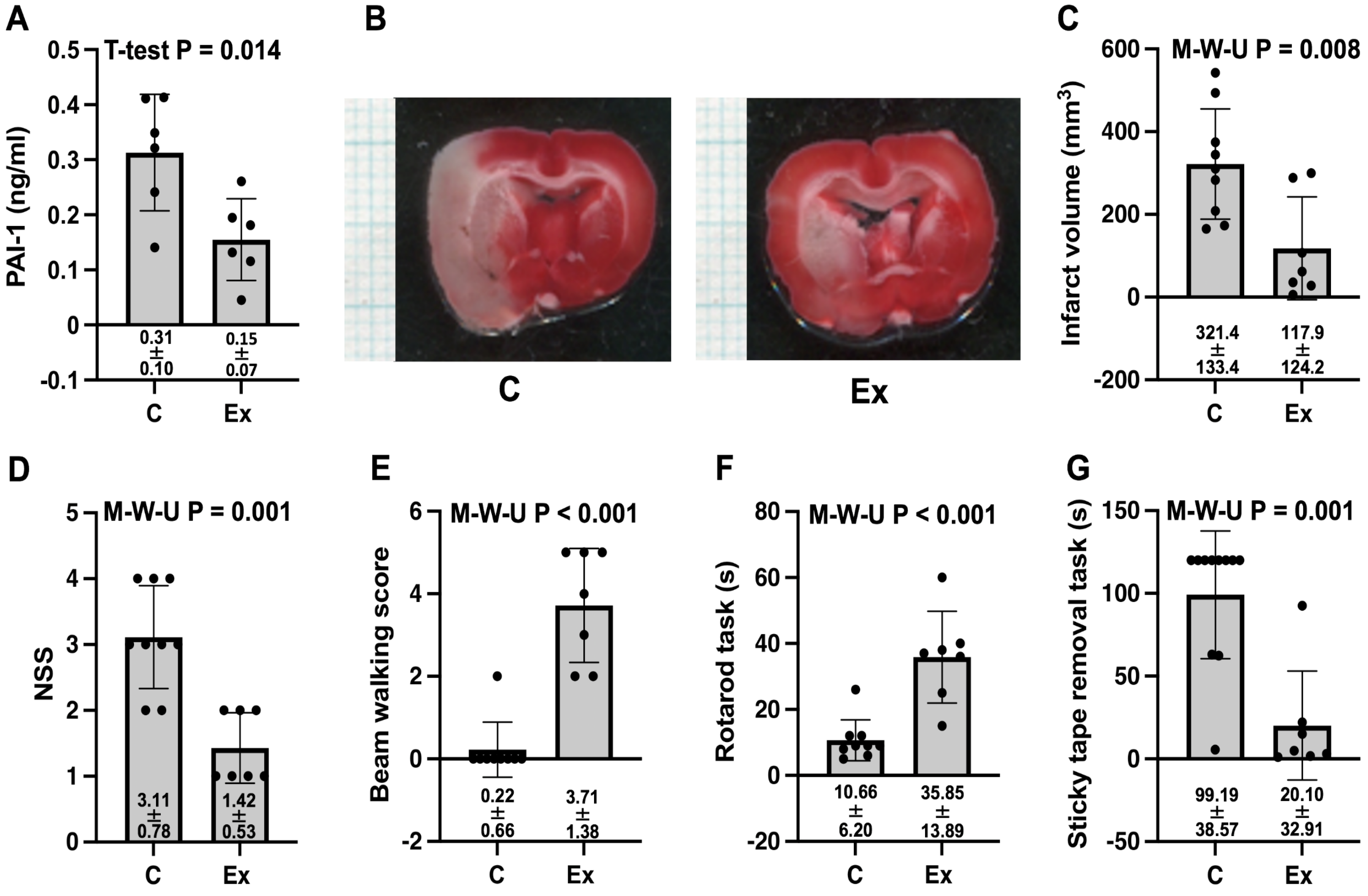

A) Concentrations of active plasminogen activator inhibitor-1 (PAI-1) were significantly lower in the exercise (Ex) group compared with the control (C) group. B) Representative 2,3,5-triphenyltetrazolium chloride (TTC)-stained cerebral sections from the C and Ex groups. C) Infarction volumes were significantly lower in the Ex group compared with the C group. D–G) NSS (D), beam walking score (E), rotarod task (F), and sticky tape-removal task (G). Data are presented as mean ± standard deviation.

Fig. S3 shows that regular preconditioning exercise before ischemic stroke significantly improved performance in the neurological severity score (NSS), beam walking score, rotarod task, and sticky tape-removal task compared with the no-exercise control condition.

M-W-U, Mann-Whitney U test.

Supplementary text: Methods and results of the animal experiments

Background

To clarify whether regular preconditioning physical activity could exert neurovascular protective effects against ischemic and hemorrhagic stroke events, the effects of preconditioning exercise needed to be tested in an animal model.

Methods

Ethics statement

This study was approved by the Kagoshima University Animal Experiment Ethics Committee and registered in the Kagoshima University Animal Experiment Protocol (approval no. MD20098, 3 February 2021; approval no. MD18078, 8 November 2018).

Animals

Forty-two male C57/BL6J mice (Japan SLC, Hamamatsu, Japan) and 33 male Sprague–Dawley rats (Charles River Laboratories Japan, Atsugi, Japan) (age: 4 weeks) were used in this study. Because similar experiments had already been conducted, and blood collection procedures may affect functional tests, studies were performed using calculations of a minimal sample size for an effect size (ES) (d) > 0.8. A total of 10 mice and 12 rats were used for blood collection, and 32 mice and 21 rats were used for functional assessment in the mice and rat experiments, respectively (Fig. S1). The animals were housed in pairs under temperature-controlled conditions (22.0 ± 1.0°C) on a 12-h light/dark cycle, with food and water available ad libitum.

Experimental protocol and preconditioning exercise

All mice and rats performed 3 days of motor-driven treadmill running (MK-680;

Muromachi Kikai, Tokyo, Japan) at speeds of 10–15 m/min and 15–25 m/min, respectively, for 10 min/day (familiarization) (Fig. S1). After familiarization, the mice and rats were randomly assigned to two groups: no exercise and hemorrhagic or ischemic strokes (C: Control group, n = 21 or 18, respectively), and exercise and hemorrhagic or ischemic stroke (Ex: Exercise group, n = 21 or 15, respectively). Before the hemorrhagic or ischemic stroke induction procedures, mice and rats in the Ex groups performed preconditioning exercise at a speed of 20 m/min or 25 m/min, respectively, for 30 min 5 days/week for 8 weeks or 3 weeks, respectively. Mice and rats in the C groups were allowed to move freely in their cage, with no additional treadmill running. After the end of the 8-week period (mice), hemorrhagic stroke was induced (mean body mass, 25.1 ± 2.0 g)[1,2]. Conversely, after the end of the 3-week period (rats), thromboembolic ischemia of the middle and posterior cerebral arteries was induced by homologous blood clot (mean body mass, 283.0 ± 30.2 g)[3,4]. Finally, the mice and rats were left to recover in their cages with sufficient food and water for 48 h and 24 h, respectively. Neurological deficits, sensorimotor function, and locomotor function were then assessed. After each test, the mice and rats were humanely euthanized, and the brain, including the ischemic region, was analyzed histologically and immunohistochemically.

Enzyme-linked immunosorbent assay (ELISA)

Collected samples were centrifuged (1500 *g* for 10 min), and plasma was frozen at −80°C and stored until just before use. For the active plasminogen activator inhibitor-1 (PAI-1) assay, a Mouse or Rat Active PAI-1 ELISA Kit (Funakoshi Co., Tokyo, Japan) was used.

Quantification of stroke

In the experimental model of acute hemorrhagic stroke, after fixation, paraffin-embedded coronal brain sections (thickness, 4 μm) were stained with Klüver–Barrer stain

(KB) for histological analyses. KB sections of the center of the injury site were viewed at ×4 magnification using a microscope (DP21; Olympus, Tokyo, Japan). The bleeding area was measured using Scion Image software version 4.0.3 (Scion Corp., Frederick, MD, USA). In the experimental model of acute ischemic stroke, after staining, sections were scanned to determine the ischemic infarct volume. Infarcts were measured using Scion Image software 4.0.3. Total infarct area (in cubic millimeters) was multiplied by the thickness of the brain sections to obtain the infarct volume.

Evaluation of neurological deficits, sensorimotor function, and locomotor function

Neurological deficits, sensorimotor function, and motor function were assessed using the beam-walking test, rotarod task, and sticky tape-removal test 48 h after hemorrhagic stroke in mice or 24 h after ischemic stroke in rats. The beam walking and rotarod task were scored for focal neurological deficits using a 28-point neurological severity score (NSS)[1], and each point was evaluated using a neurological grading system with a 5-point score (0–4). In the motor behavior test, animals were examined using a beam-walking task using an elevated narrow beam (mice: 30 cm long × 1 cm wide; rats: 100 cm long × 2.5 cm wide). The time to traverse the beam was recorded and analyzed across three trials per day (maximum trial duration, 60 s). The beam-walking task was graded with a 6-point score (0–5). Motor function and balance were evaluated using the rotarod task (MK-670; Muromachi Kikai Co., Tokyo, Japan). Each animal was placed on the rotarod cylinder, and the duration that the animal remained on the cylinder was measured. The rod (mice: 3 cm in diameter; rats: 10 cm in diameter) was covered with smooth rubber. For the mice, rotation speed was increased from 0 to 40 rpm in 4-rpm increments every 6 s. For the rats, rotation speed was increased from 0 to 25 rpm in 2.5-rpm increments every 6 s. The trial ended when the animal fell off the cylinder. Each animal performed three trials. The best latency until fall for each animal was used for the analysis. Sensorimotor dysfunction was assessed using the adhesive sticky tape-removal test

(sticky labels: mice: 6.1 mm × 10 mm; rats: 12.5 mm × 12.5 mm), as previously described[5]. The time required to remove the label from the forelimbs was recorded across two trials for each forepaw, with the best recorded time of the two trials used for the analysis.

Statistical analyses

The independent t test and Mann–Whitney U test were used as appropriate for between-group analyses. Fisher's exact probability test was also used to analyze associations between exercise and mortality after modeling.

RESULTS

Hemorrhagic stroke model in mice

PAI-1 levels were lower in the Ex vs. C groups, indicating that PAI-1 decreased by approximately 50% and preconditioning exercise enhanced fibrinolysis. Importantly, two of 16 mice in the C group died compared with 0 of 16 mice in the Ex group ($P = 0.484$). The hemorrhage volume was larger in the Ex vs. C groups. The NSS scores were significantly better in the Ex vs. C groups; however, the beam walking scores and walking times in the rotarod task were similar. The right limbs (affected side) showed significantly better latencies in the Ex vs. C groups, indicating that regular preconditioning exercise partially improved motor or sensory neurological functional deficits (Fig. S2).

Ischemic stroke model in rats

PAI-1 levels were significantly lower in the Ex vs. C groups by approximately half, indicating that preconditioning exercise enhanced fibrinolysis. One of the 12 rats died in the C group, and two of the nine rats died in the Ex group ($P = 0.999$). A significant difference in infarct volume was identified between the Ex and C groups, indicating that the cerebral infarct volume decreased more in the Ex group vs. the C group. Significant

differences in neurological scores, beam walking scores, walking times in the rotarod task, and times in the sticky tape-removal task were also found, indicating that motor or sensory neurological functional deficits improved notably with exercise (Fig. S3).

References

1 Clark W, Gunion-Rinker L, Lessov N, Hazel, K. Citicoline treatment for experimental intracerebral hemorrhage in mice. Stroke. 1998;29:2136-40.

2 Wang J, Rogove AD, Tsirka AE, Tsirka SE. Protective role of tuftsin fragment 1-3 in an animal model of intracerebral hemorrhage. Ann Neurol. 2003;54,655-64.

3 Kikuchi K, Setoyama K, Kawahara KI, Nagasato T, Terashi T, Ueda K, et al. Edaravone, a synthetic free radical scavenger, enhances alteplase-mediated thrombolysis. Oxid Med Cell Longev. 2017;6873281.

4 Kikuchi K, Setoyama K, Tanaka E, Otsuka S, Terashi T, Nakanishi K, et al. Uric acid enhances alteplase-mediated thrombolysis as an antioxidant. Sci Rep. 2018;8:15844.

5 Sughrue ME, Mocco J, Komotar RJ, Mehra A, D'Ambrosio AL, Grobelny BT, et al. An improved test of neurological dysfunction following transient focal cerebral ischemia in rats. J Neurosci Methods. 2006;151:83-9.
